# UAV-Enabled Maritime IoT D2D Task Offloading: A Potential Game-Accelerated Framework

**DOI:** 10.3390/s25185820

**Published:** 2025-09-18

**Authors:** Baiyi Li, Jian Zhao, Tingting Yang

**Affiliations:** 1 Navigation College, Dalian Maritime University, Dalian 116026, China; libaiyi@dlmu.edu.cn (B.L.); yangtingting820523@163.com (T.Y.); 2 Department of Network Intelligence, Peng Cheng Laboratory, Shenzhen 518000, China

**Keywords:** maritime IoT systems, task offloading, USV, device-to-device communications, game theory

## Abstract

Maritime Internet of Things (IoT) with unmanned surface vessels (USVs) faces tight onboard computing and sparse wireless links. Compute-intensive vision and sensing workloads often exceed latency budgets, which undermines timely decisions. In this paper, we propose a novel distributed computation offloading framework for maritime IoT scenarios. By leveraging the limited computational resources of USVs within a device-to-device (D2D)-assisted edge network and the mobility advantages of UAV-assisted edge computing, we design a breadth-first search (BFS)-based distributed computation offloading game. Building upon this, we formulate a global latency minimization problem that jointly optimizes UAV hovering coordinates and arrival times. This problem is solved by decomposing it into subproblems addressed via a joint Alternating Direction Method of Multipliers (ADMM) and Successive Convex Approximation (SCA) approach, effectively reducing the time between UAV arrivals and hovering coordinates. Extensive simulations verify the effectiveness of our framework, demonstrating up to a 49.6% latency reduction compared with traditional offloading schemes.

## 1. Introduction

Shipping occupies a crucial position within the national transportation systems of various countries, delivering significant economic and social benefits [1]. With the rapid development of intelligent shipping and digitalization, maritime IoT systems have recently emerged as a key enabler for enabling real-time vessel monitoring, environmental sensing, and autonomous navigation [2,3,4]. However, unlike terrestrial IoT, maritime IoT networks face unique challenges, including sparse wireless connectivity, harsh propagation environments, and the limited onboard computation capacity of USVs [5,6]. At the same time, computation-intensive tasks such as vision-based navigation, image recognition, and safety-critical decision-making involve processing substantial volumes of raw data, and the computational latency incurred may exceed the permissible thresholds [7]. These factors highlight the necessity of task offloading networks, where USVs can offload workloads to nearby assisting vessels or UAV-assisted edge nodes to ensure efficient and timely task execution.

Mobile edge computing (MEC), built upon 5G/6G wireless communication technologies, offers a promising solution [8]. MEC addresses this challenge by pushing abundant computational resources from cloud servers to the network edge, thereby meeting the escalating computational and communication demands of USVs. However, constrained by the inherent limitations of the hardware capabilities of MEC platforms themselves, MEC systems currently cannot provide on-demand, ubiquitous temporary services within hotspot areas. Compared to traditional MEC, UAV-assisted MEC offers the capability to rapidly establish edge computing nodes in areas lacking fixed infrastructure, thereby overcoming the spatial constraints inherent in conventional edge computing deployments. Furthermore, UAVs can dynamically adjust their positions in the airspace, enabling broader coverage and enhanced proximity to task locations, which significantly improves computational efficiency and service quality.

The inherent dynamic resource scheduling capability of UAV-assisted systems allows for optimization of the computational resource distribution based on task demands, thereby enhancing system adaptability and resilience. In [9], the authors integrate bandwidth allocation and UAV positioning to minimize the task delay. In [10], the authors optimize the task offloading ratio by establishing a two-tier architecture consisting of users and UAVs. In [11], the authors formulate a UAV energy minimization problem by comprehensively considering the USVs’ task execution methods, UAV trajectories, UAV arrival times, and UAV hovering coordinates. In [12], the authors proposed approach involves a USV-UAV cooperative platform where UAVs dynamically position themselves and land on USVs with low latency to perform tasks. Another proposal introduces a UAV-assisted maritime IoT network, enabling USVs to offload computationally intensive tasks to UAVs [13]. Although UAV-assisted MEC pushes computational resources from the remote cloud towards the network edge, resource contention arises when multiple USVs simultaneously require task offloading to the edge cloud. This competition for resources directly impacts user experience.

D2D communication, enabling direct data transmission between adjacent users, has garnered significant academic interest for its potential application as an auxiliary approach to task offloading. In [14], the authors discussed the potential of D2D communication for task offloading, aiming to maximize the number of users capable of completing computational tasks through optimized D2D link establishment. However, most existing research suffers from two common limitations. Firstly, there is a prevalent assumption that stable D2D communication links are pre-established. In practice, constrained by factors such as physical distance and social ties, certain mobile users struggle to establish stable D2D links. This foundational assumption deviates from real-world scenarios, necessitating in-depth investigation into effective D2D pairing mechanisms. Secondly, existing research has predominantly focused on individual optimization dimensions, such as channel allocation, D2D pairing, and task offloading modes, ignoring the strong coupling relationships among these three aspects.

Game theory provides an effective theoretical foundation for multi-agent resource allocation, incentive mechanism design, and system-wide collaborative optimization. The construction of suitable game-theoretic models enables the capture of the evolution of each participant’s strategy under mutual influence, thereby helping the system maximize overall utility or payoff under limited resources. In [15,16], game theory is used to manage communication resources. By modeling the channel selection problem as an exact potential game (EPG), a balance between delay and energy consumption is achieved. In [17], a stochastic congestion game based on an EPG was proposed to investigate the load balancing issue in MEC, aiming to minimize the delay of task execution. In [18], an EPG is used for federated split learning. On the basis of BFS, distributed machine learning with low latency costs is realized through an EPG and resource optimization. In [19], the authors model the decision process in task offloading and resource allocation as a potential game, which achieves efficient, distributed task offloading and resource allocation with a convergence guarantee in a large-scale U-MEC network.

To integrate the aforementioned technological advantages while ensuring alignment with practical shipping application scenarios, we design an accelerated D2D distributed computing offloading framework based on EPG theory, called ADTO. This framework aims to achieve efficient task execution by leveraging the high-mobility and cluster head (CH) [20,21] characteristics of UAVs in UAV-assisted edge networks and the constrained computational resources in D2D-assisted edge networks [22]. Specifically, the proposed framework employs potential game theory to model the client clustering optimization problem as a distributed computation offloading game. This approach aims to maximize the overall offloading efficiency while simultaneously addressing communication limitations and enhancing resource utilization. Building upon this, we formulate a global latency minimization problem that jointly optimizes UAV hovering coordinates and arrival times. This problem is solved by decomposing it into subproblems addressed via a joint ADMM and SCA approach, effectively reducing the time between UAV arrivals and hovering coordinates. The main contributions of this paper can be outlined as follows:

- This paper introduces a novel edge computing architecture in maritime IoT systems to fully utilize the advantage of UAV-assisted edge computing (with UAVs serving as dynamic CHs) and D2D-assisted edge networks (with USVs cooperating in clusters), aiming to provide USVs with low-latency and reliable computing services.
- A global task offloading latency minimization model is constructed by jointly optimizing D2D link selection, UAV arrival time, and hovering coordinates. To reduce computational complexity, a heuristic solution is proposed to decompose the proposed problem into multiple subproblems and design suboptimal solutions, thereby reducing the optimization cost associated with long-term repeated optimization.
- The simulation results under simulated realistic scenarios and various system settings demonstrate that our proposed framework can effectively reduce the overall system delay while making full use of the available communication and computing resources.

The rest of this paper is organized as follows: Section 2 mathematically models the global delay issue and presents an overview of the proposed ADTO algorithm. Section 3 presents a solution to the problem mentioned in Section 2. Section 4 presents the results of the experiment. Section 5 offers a conclusion to and discussion on this essay.

## 2. System Overview and Problem Formulation

### 2.1. System Model

The UAV-assisted D2D edge network under consideration comprises multiple distributed USVs possessing heterogeneous computational and communication resources, multiple UAVs equipped with identical communication resources, and a single terrestrial base station (TBS) acting as an edge server with abundant computational resources. Furthermore, the TBS is equipped with a global controller capable of perceiving USV positions, UAV computational and energy states, and a priori Channel State Information (CSI). Each USV can establish D2D links with neighboring USVs. However, due to constraints imposed by geographical factors and hardware limitations, USVs cannot establish direct communication links with the TBS; consequently, UAVs act as communication relays between the TBS and USVs by establishing wireless cellular links with USVs and forming Line of Sight (LoS) connections with the TBS. Specifically, the distributed USVs are categorized into two types: task clients (TCs) and assisting clients (ACs). There are *L* TCs capable of participating in task offloading by contributing their local tasks, represented as L={1,2,…,L}, and *C* ACs located near TCs that can assist TCs in task completion by providing spare computational resources, represented as C={1,2,…,C}. Therefore, each TC can form a collaborative cluster with multiple ACs via D2D communication to perform task offloading, thereby overcoming bottlenecks caused by limited computational or communication resources, with the *L* TCs clustered into *L* collaborative clusters. To simplify the analysis, we assume each cluster contains exactly one TC. Without loss of generality, cluster l∈L is associated with a total of $n_{l}$ ACs, denoted by $N_{l}=\left\{0,1,\ldots ,n_{l}\right\}$. When $N_{l}=0$, it denotes the index of the TBS. Within a three-dimensional Cartesian coordinate system, UAVs are assumed to fly and hover at a fixed altitude *H*; when UAV *l* serves USV *i* located at coordinates $q_{i}=\left(x_{i},y_{i},0\right)$, it has a corresponding hovering coordinate $q_{l}=\left(x_{l},y_{l},H\right)$. To simplify the analysis and prevent resource sharing conflicts among ACs, we assume each AC can join at most one cluster. Let the binary variable $v_{l}$ indicate the association status between AC *l* and cluster *l*, where $V_{l}=1$ if AC *l* is selected by cluster *l*, and $V_{l}=0$ otherwise. Finally, we can derive

(1) $$\sum_{l=1}^{L}V_{l,c}\leq 1,\forall c\in C.$$

The proposed method aims to collaboratively complete tasks by splitting a task with a linear topology into multiple subtasks within each learning cluster and assigning these subtasks to cluster members based on their computational capabilities. For simplicity, the tasks considered in this paper can be divided into up to the total number of clients within a cluster. Each task contains *D* segmentable layers, denoted as D={1,2,…,D}. Let the binary variable $z_{l,d}$ indicate the splitter selection status for segmentable layer d∈D, where $z_{l,d}=1$ signifies that segmentation layer *d* is selected, and $z_{l,d}=0$ otherwise. Therefore, the task allocation corresponding to cluster *l* can be denoted as the vector $z_{l}=\left\{z_{l,1},z_{l,2},\ldots ,z_{l,D}\right\}$. For simplicity, each cluster member is restricted to forwarding its model execution output solely to its immediate successor within the chain. Therefore, the USV clustering and task assignment strategies should adhere to the following constraints:

(2) $$\sum_{d=1}^{D}z_{l,d}⩽D,\forall l\in L.$$

To facilitate further analysis of this scenario, we introduce the definitions as follows.

**Definition** **1** (Connected graph)**.** *For any graph G=(V,E), let (V) denote the set of nodes, representing the set of USVs, and let (E) denote the set of edges, representing the wireless communication links established between these nodes. If any two distinct vertices in V are connected, the graph G is referred to as a connected graph.*

### 2.2. Problem Formulation

Based on the discussion in the previous section, the latency incurred by the distributed task offloading framework proposed in this paper comprises two components: intra-cluster task execution delay and UAV flight delay. This subsection provides a detailed analysis of the latency contributions from each component.

**Global Task Distribution:** When the computation tasks commence, the TBS broadcasts the global task to the CH of each cluster. The downlink data transfer rate from TBS to CH *l* in a cluster can be expressed as

(3) $$r_{l}^{DL}=B_{l}\log_{2}\left(1+\frac{ph_{l,0}}{\sigma^{2}d_{0,l}^{2}}\right),$$

where $B_{l}\left(0<B_{l}\right)$ denotes the radio bandwidth allocated by the TBS to the UAV serving as the CH, *p* denotes the transmission power of the TBS, $\sigma^{2}$ represents the noise variance, $d_{0,l}=\sqrt{\|q_{l}-q_{0}{\|}^{2}+H^{2}}$ signifies the distance between the TBS and the UAV *l* hovering coordinate, *p* is the transmit power of the TBS, and $h_{l.0}$ represents the channel power gain between the UAV and the TBS. The transmission delay from the TBS to the CH of each cluster can be expressed as

(4) $$t_{l}^{DL}=\frac{w_{l}}{R_{l}^{DL}},$$

where $w_{l}$ represents the data size of a global task, which is assumed to be identical for all clusters.

**Intra-Cluster Task Processing:** Intra-cluster task execution consists of three primary sub-phases: intra-cluster task distribution, the task execution process, and task collection by the CH. For analytical convenience, we discuss the process using cluster *l* as a representative example.

- *Intra-Cluster Task Distribution:* Within the cluster, the designated UAV serving as the CH acts as the communication relay. USVs access the CH via a wireless cellular link. Consequently, the intra-cluster task distribution latency from the CH to USV *i* can be expressed as
  (5) $$t_{a,i}=\frac{w_{l,i}}{r_{i}},$$
  where $r_{i}=B\log_{2}\left(1+\frac{ph_{l,0}}{\sigma^{2}\left(\right\|q_{i}-q_{l}{\|}^{2}+H^{2})}\right)$ represents the data transmission rate between the CH and USV *i*, measured in bits per second, and $w_{l,i}$ denotes the size of the task processed by USV *i*.
- *The Task Execution Process:* Once intra-cluster task allocation is completed, USV *i* immediately executes its assigned subtask. Therefore, the task execution delay of USV *i* can be expressed as
  (6) $$t_{l,i}^{comp}=\frac{C_{i}w_{l,i}}{f_{i}},$$
  where $C_{i}$ represents the number of CPU cycles required to process a data sample, and $f_{i}$ denotes the CPU computation frequency of USV *i*.
  Let $w_{l,i}^{id}$ denote the intermediate data forwarded from USV *i* to USV *j* after local computation. The corresponding communication delay is
  (7) $$t_{l,i}^{comm}=\frac{w_{l,i}^{id}}{r_{i,j}},$$
  where $r_{i,j}$ denotes the D2D data transmission rate between USV *i* and USV *j*.
- *Task Collection by the CH:* Upon the completion of execution, the CH instructs the USVs within the cluster to return their processed segments for aggregation. Since the task collection process is almost identical to the intra-cluster task distribution process, the delay of model collection $t_{c,i}$ is equal to $t_{a,i}$.
  Finally, the total intra-cluster processing delay can be calculated as
  (8) $$t_{l,i}^{TTE}=2t_{a,i}+t^{TE},$$
  where $t^{TE}=t_{l,i}^{comm}+t_{l,i}^{comp}$.

**Local Subtask Uploading:** Upon task completion, the CH uploads the aggregated result to the TBS. The transmission rate from the CH to the TBS can be calculated as

(9) $$r_{l}^{UL}=B_{l}\log_{2}\left(1+\frac{p_{l}h_{l}}{\sigma^{2}d_{l,0}^{2}}\right),$$

where $p_{l}$ denotes the transmission power of the CH. The corresponding task upload delay from the CH to the TBS can be expressed as

(10) $$t_{l}^{UL}=\frac{w_{l}}{r_{l}^{UL}}.$$

Finally, under the USV clustering strategy and task allocation strategy, the overall delay of the cluster can be expressed as

(11) $$T_{l}=t_{l}^{DL}+t_{l,i}^{TTE}+t_{l}^{UL}.$$

**The UAV Time Cost Model:** We assume the propulsion power of each UAV is independent of external factors and solely dependent on its flight velocity $v_{l}$. The time consumed for a UAV to travel between any two hovering coordinates associated with USVs can be derived as

(12) $$t_{l(i,j)}^{f}=\frac{\|q_{j}-q_{i}\|}{v_{l}}.$$

Let the binary variable $\rho_{l(i,j)}$ indicate whether UAV *l* travels from hovering coordinate *i* to hovering coordinate *j*, where $\rho_{l(i,j)}=1$ signifies that UAV *l* flies from point *i* to point *j*, and $\rho_{l(i,j)}=1$ otherwise. When i=0 or j=0, it denotes the index of the TBS. Given that UAV *l* must depart from the TBS and ultimately return to it, we derive the following constraints:

(13) $$\sum_{i=0}^{n_{l}}\rho_{l(0,i)}=\sum_{g=0}^{n_{l}}\rho_{l(j,0)}=1,\forall l\in L.$$

Due to the sequential dependency of D2D computation offloading subtasks, UAV *l* can serve USV *j* if and only if it has successfully completed service to USV *i*. Thus, the constraint can be derived as follows:

(14) $$\sum_{i=0}^{n_{l}}\rho_{l(i,j)}=\sum_{g=0}^{n_{l}}\rho_{l(j,g)}=1,\forall l\in L,g\neq i,j.$$

It is noteworthy that UAV *l* must remain in a hovering state until USV *i* completes the execution of its allocated subtask. This requirement leads to the following constraint

(15) $$t_{l,i}^{a}+T_{l}+t_{l(i,j)}^{f}\leq t_{l,j}^{a},i,j\in N_{l},l\in L,$$

where $t_{l,i}^{a}$ is the time when UAV *l* reaches the hovering coordinates of USV *i*.

Subject to the maximum battery capacity $E_{l}^{max}$ of the UAV, let $T_{l}^{max}$ denote its maximum allowable service time. This imposes the following constraint:

(16) $$\sum_{i=0}^{n_{l}}\sum_{j=0}^{n_{l}}\rho_{l(i,j)}\left(T_{l}+t_{l(i,j)}^{f}\right)\leq t_{l}^{\max}.$$

Considering the limited computational resources of the USVs and the limited communication resources of the TBS, this paper globally optimizes the USV clustering scheme, the task allocation strategy, and the arrival time and hovering coordinates of each UAV to minimize the total delay cost of each TC. The problem can be formulated as a total latency cost minimization problem, with the objective and constraints described as follows:

(17) $$\begin{array}{cc} \min_{\left\{n_{l},s_{l},t,q_{l}\right\}}: & T=\sum_{l=1}^{L}\sum_{i=0}^{n_{l}}\sum_{j=0}^{n_{l}}\rho_{l(i,j)}\left(T_{l}+t_{l(i,j)}^{f}\right)\\ s.t. & (1),(2),(13)–(16). \end{array}$$

## 3. Problem Decomposition and Proposed Method

**Figure 1:**
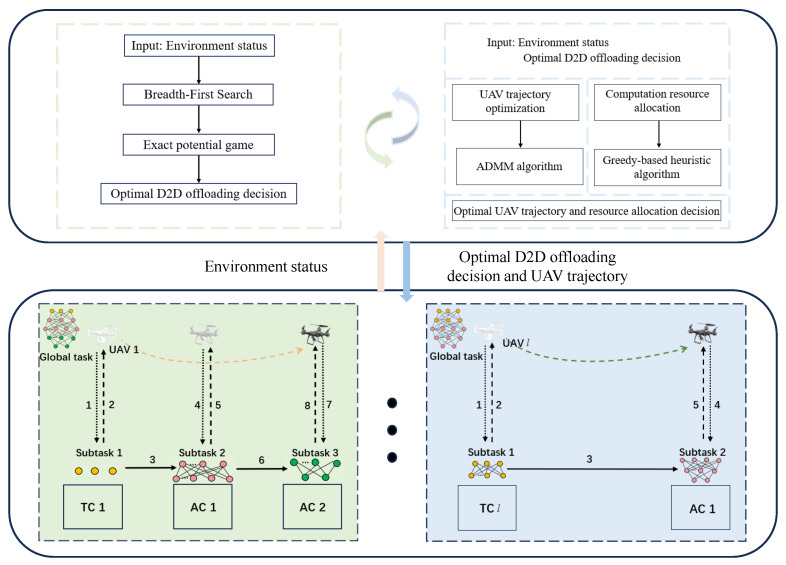
Illustration of proposed problem decomposition and solution approach.

The problem formulated in the preceding section involves three tightly coupled subproblems: the USV clustering strategy, the task allocation strategy, UAV arrival time optimization, and hovering coordinate optimization. For instance, the task allocation strategy inherently depends on the established client clustering strategy. This tight coupling results in the exponential complexity evident in (17), substantially increasing the computational costs. To address this challenge, as shown in Figure 1, we first propose a client clustering game based on BFS and EPG theory. Subsequently, we efficiently optimize the task allocation strategy and UAV hovering coordinate problem utilizing a greedy algorithm combined with the ADMM. When the TBS receives the current environment state as a global controller, the current optimal UAV flight trajectory and the optimal D2D offloading decision are calculated by running the proposed scheme and fed back to the USV and UAV in the environment.

### 3.1. BFS-Based Distributed Game Clustering

**Figure 2:**
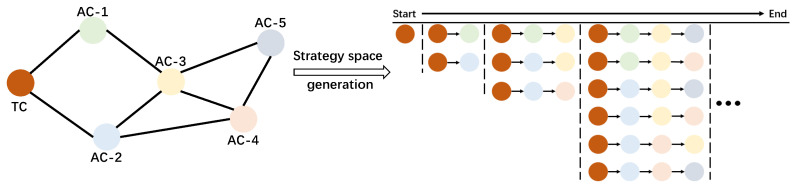
Illustration of BFS-based distributed game clustering.

Each learning cluster seeks a local strategy that minimizes its own latency. Accordingly, the client-clustering task is modeled as a multi-player game in which *L* clusters serve as players. The strategy of cluster *l* is denoted by $s_{l}$, with its feasible strategy set represented by $S_{l}$. Here, we generate the game’s strategy space by applying BFS. Specifically, as depicted in Figure 2, starting from the origin TC, BFS prioritizes exploring all neighboring ACs before proceeding to explore ACs at the next level further away, proceeding level by level until the target is found or all reachable ACs have been traversed.

We assume that communication state variations within each time window are relatively stable. Under this distributed game framework, each agent considers not only its own latency but also the impact of its actions on the overall network state. Specifically, for each TC, a clustering strategy $s_{l}$ can be determined. The joint strategy of *L* clusters can then be represented as $s=\{s_{1},s_{2},\ldots ,s_{L}\}$. Notably, $s_{-l}$ denotes the clustering strategies of all clusters except cluster *l*. Within this context, we employ marginal utility theory [23,24] to evaluate the overall impact of a strategy. Furthermore, aligning with the optimization objective in (17), the utility function for a cluster is defined as

(18) $$A_{l}\left(s_{l},\;s_{-l}\right)\;=\;-\phi_{l}\left(s_{l},\;s_{-l}\right)+\;\sum_{j\neq l}[\left(\;-\phi_{j}\left(s_{j},\;s_{-j}\right)\;+\;\phi_{j}\left(s_{j},\;s_{-j\setminus l}\right)\right],$$

where $-\phi_{j}\left(s_{j},s_{-j\setminus l}\right)$ denotes the utility of cluster *j* under the condition that cluster *l* does not take any action. Therefore, $\sum_{j\neq l}[\left(-\phi_{j}\left(s_{j},s_{-j}\right)+\phi_{j}\left(s_{j},s_{-j\setminus l}\right)\right]$ signifies the change in the aggregate utility of the other clusters induced by the strategic action of cluster *l*.

The objective of the distributed clustering game is to minimize cluster conflicts and maximize each cluster’s utility, which critically depends on the existence of a Nash Equilibrium (NE) within this distributed clustering game framework.

**Definition** **2** (Nash Equilibrium [25])**.** A joint strategy $s^{*}=\left\{s_{1}^{*},\ldots ,s_{L}^{*}\right\}$ is called an NE of the formulated game if and only if, holding all other clusters’ strategies fixed, no single cluster can improve its utility by unilaterally altering its own strategy. In other words,

(19) $$A_{l}\left(s_{l}^{*},s_{-l}^{*}\right)\geq A_{l}\left(s_{l},s_{-l}^{*}\right),\forall l\in L,s_{l}\in S_{l}.$$

To ensure that the proposed game-based method possesses an NE and achieves convergence, we introduce the concept of the EPG and further explore the existence of an NE under its theoretical framework.

**Definition** **3** (EPG [26])**.** *If an exact potential function $\psi (s_{l},s_{-l})$ exists that satisfies the following conditions, then the game is called an EPG:*

(20) $$A_{l}\left(s_{l}^{\prime },s_{-l}\right)-A_{l}\left(s_{l},s_{-l}\right)=\psi \left(s_{l}^{\prime },s_{-l}\right)-\psi \left(s_{l},s_{-l}\right),$$

*where $s_{l}^{\prime },s_{-l}\in S_{l}$.*

**Theorem** **1.**  *A distributed USV clustering game is an EPG if and only if at least one pure-strategy NE point exists.*

**Proof.**  Based on the optimization problem, we can define an exact potential function that satisfies the following conditions:

(21) $$\mu \left(s_{l},s_{-l}\right)=\sum_{l=1}^{L}-\psi_{l}\left(s_{l},s_{-l}\right).$$

The potential function is essentially the sum of all groups’ payoffs across the entire network. Suppose that the decision of group *l* changes from $s_{l}$ to $s_{l}^{\prime }$. The change in its utility function is presented in (22).

(22) $$\begin{array}{cc} \; & \;\;A_{l}\left(s_{l}^{\prime },s_{-l}\right)\;\;-\;\;A_{l}\left(s_{l},s_{-l}\right)\\ \; & =\;\;-\phi_{l}\left(s_{l}^{\prime },s_{-l}\right)\;\;+\;\;\sum_{j\neq l}\left(-\phi_{j}\left(s_{j},s_{-j}^{\prime }\right)\;\;+\;\;\phi_{j}\left(s_{j},s_{-j\setminus l}^{\prime }\right)\right)\;\;-\;\;\left(\;\;-\phi_{l}\left(s_{l},s_{-l}\right)\;\;+\;\;\sum_{j\neq l}\left(-\phi_{j}\left(s_{j},s_{-j}\right)\;\;+\;\;\phi_{j}\left(s_{j},s_{-j\setminus l}\right)\right)\;\;\right)\\ \; & =\;\;-\phi_{l}\left(s_{l}^{\prime },s_{-l}\right)\;\;+\;\;\phi_{l}\left(s_{l},s_{-l}\right)\;\;+\;\;\sum_{j\neq l}\left(-\phi_{j}\left(s_{j},s_{-j}^{\prime }\right)\;\;+\;\;\phi_{j}\left(s_{j},s_{-j}\right)\right)\;\;-\;\;\sum_{j\neq l}\left(-\phi_{j}\left(s_{j},s_{-j\setminus l}\right)+\phi_{j}\left(s_{j},s_{-j\setminus l}^{\prime }\right)\right) \end{array}$$

Although the behavior of cluster *l* has changed, if cluster *l* abandons its decision while other clusters maintain their own decisions, the absence of cluster *l*’s action would have the same impact on the other clusters (i.e., $\psi_{j}\left(s_{j},s_{-j\setminus l}\right)=\psi_{j}\left(s_{j},s_{-j\setminus l}^{\prime }\right)$). Thus, we can ignore the third term in (18) and reorganize the above expression, as shown in (23).

(23) $$\begin{array}{cc} \;\;\;\;A_{l}\left(s_{l}^{\prime },s_{-l}\right)-A_{l}\left(s_{l},s_{-l}\right) & \;\;=\;\;-\phi_{l}\left(s_{l}^{\prime },s_{-l}\right)\;\;+\;\;\phi_{l}\left(s_{l},s_{-l}\right)\;\;+\;\;\sum_{j\neq l}\left(-\phi_{j}\left(s_{j},s_{-j}^{\prime }\right)\;\;+\;\;\phi_{j}\left(s_{j},s_{-j}\right)\right)\\ \; & \;\;=\;\;-\phi_{l}\left(s_{l}^{\prime },s_{-l}\right)\;\;+\;\;\sum_{j\neq l}-\phi_{j}\left(s_{j},s_{-j}^{\prime }\right)\;\;-\;\;\left(-\phi_{l}\left(s_{l},s_{-l}\right)+\sum_{j\neq l}-\phi_{j}\left(s_{j},s_{-j}\right)\right)\\ \; & \;\;=\;\;\psi \left(s_{l}^{\prime },s_{-l}\right)\;\;-\;\;\psi \left(s_{l},s_{-l}\right) \end{array}$$

Therefore, any unilateral change in a cluster’s decision results in an identical variation in both its individual utility and the potential function, confirming that the proposed distributed task offloading game is an EPG. According to the fundamental properties of an EPG, at least one pure-strategy NE exists, thus completing the proof of Theorem 1. □

### 3.2. Optimizing the Task Allocation for a Given Clustering Strategy

We propose a greedy-based heuristic algorithm that proportionally assigns computational loads based on each USV’s resources. UAVs within clusters execute a zero-workload virtual segment, ensuring feasibility of task allocation. The detailed procedure is presented in Algorithm 1.
**Algorithm** **1** Task offloading algorithm for given clustering strategy.**Input:** Clustering strategy: $s_{l}$; computing capability of each USV: $\left\{f_{1},f_{2},\ldots ,f_{n_{l}+1}\right\}$; transmission rates between nodes: $\left\{r_{1,2},r_{2,3},\ldots ,r_{n_{l},n_{l}+1}\right\}$; computational workload of all *D* layers: $\left\{w_{1},w_{2},\ldots ,w_{D}\right\}$; transmitted intermediate data size between corresponding *M* layers: $\left\{w_{1,2}^{id},w_{2,3}^{id},\ldots ,w_{D-1,D}^{id}\right\}$.
**Output:** Task splitting and allocation strategy: $z_{l}$;
1:Initialize the last selected split layer d=0;2:Initialize the computation workload of each USV *i* as $w_{i}=\frac{f_{i}\sum_{d=1}^{D}w_{d}}{\sum_{i=1}^{n_{l}+1}f_{i}}$;3:Initialize the initial communication cost of each USV *i* as $comm_{i}=\frac{w_{d,d+1}^{id}}{r_{i,i+1}},i\in N_{l},d\in D$;4:**for** each USV $i=1,\ldots ,n_{l}$ **do**5:   Calculate computational workload from *d* to $d^{\prime }$;6:   **while** $\sum_{d}^{d^{\prime }}w_{d}\approx w_{i}$ **do**7:      Update $d=d^{\prime }$, $k_{l,m}\leftarrow 1$;8:      Update $comm_{i}$;9:   **end while**10:**end for**


### 3.3. Optimizing UAV Arrival Times and Hoverings Coordinate for a Given Clustering Strategy

To address the problem of optimizing UAV arrival times and hovering coordinates given fixed client clustering and task allocation strategies, we decouple and reformulate the problem into dedicated subproblems for optimization.

**Optimizing UAV Arrival Time** *t*: For any feasible $q_{l}$, (17) can be reduced to

(24) $$\begin{array}{cc} \min_{\left\{t\right\}}: & T=\sum_{l=1}^{L}\sum_{i=0}^{n_{l}}\sum_{j=0}^{n_{l}}\rho_{l(i,j)}\left(T_{l}+t_{l(i,j)}^{f}\right)\\ s.t. & (15),(16). \end{array}$$

Obviously, (24) constitutes a convex optimization problem with respect to the arrival time *t*. Consequently, we can efficiently solve it using the method of Lagrange multipliers. Having determined when the UAV arrives at the task point, the uncertainty surrounding its hovering coordinate $q_{l}$ is reduced, thereby simplifying subsequent optimization stages.

**Optimizing UAV Hovering Coordinates** $q_{l}$: After obtaining a feasible solution for the UAV arrival time according to (24), the optimization problem for UAV hovering coordinates can be expressed as follows.

(25) $$\begin{array}{cc} \min_{\left\{q_{l}\right\}}: & T=\sum_{l=1}^{L}\sum_{i=0}^{n_{l}}\sum_{j=0}^{n_{l}}\rho_{l(i,j)}\left(T_{l}+t_{l(i,j)}^{f}\right)\\ s.t. & (15),(16). \end{array}$$

Due to the nonlinear relationship between $T_{l}$, $t_{l(i,j)}^{f}$, and the UAV hovering coordinate $q_{l}$, (25) is rendered a non-convex problem. To address this, we employ the ADMM for its solution. Specifically, we first reformulate 25 into an ADMM-compliant form and then decompose it into a set of subproblems that can be efficiently solved in parallel. To render (25) separable, we introduce the following two auxiliary variables, namely $q_{l}^{*}$ and $q_{l}^{\prime }$, such that

(26) $$q_{l}^{*}=q_{l},q_{l}^{\prime }=q_{l}.$$

Building upon (25), constraints (15) and (16) can be reformulated, after substituting $q_{l}$, as

(27) $$t_{l,i}^{a}+T_{l}^{*}+t_{l(i,j)}^{*f}\leq t_{l,j}^{a},i,j\in N_{l},l\in L,$$

(28) $$\sum_{i=0}^{n_{l}}\sum_{j=0}^{n_{l}}\rho_{l(i,j)}\left(T_{l}^{\prime }+t_{l(i,j)}^{\prime f}\right)\leq t_{l}^{\max}.$$

Therefore, (25) can be reformulated in the equivalent ADMM form as follows:

(29) $$\begin{array}{cc} \min_{\left\{q_{l},q_{l}^{*},q_{l}^{\prime }\right\}}: & T=\sum_{l=1}^{L}\sum_{i=0}^{n_{l}}\sum_{j=0}^{n_{l}}\rho_{l(i,j)}\left(T_{l}+t_{l(i,j)}^{f}\right)\\ s.t. & (26)–(28). \end{array}$$

Through the introduction of auxiliary variables, constraints (27) and (28) are decoupled, enabling the problem to be decomposed. Following decoupling, the application of the ADMM allows each subproblem to be computed in parallel, thereby enhancing the computational efficiency. Here, we construct the augmented Lagrangian function with an additional quadratic penalty term to accelerate the convergence and improve the numerical stability, as follows:

(30) $$\begin{array}{cc} \; & L\left(q_{l},q_{l}^{*},q_{l}^{\prime },\omega_{1},\omega_{2}\right)=\sum_{l=1}^{L}\sum_{i=0}^{n_{l}}\sum_{j=0}^{n_{l}}\rho_{l(i,j)}\left(T_{l}+t_{l(i,j)}^{f}\right)\\ \; & +\frac{\upsilon_{1}}{2}\|q_{l}^{*}-q_{l}+\omega_{1}{\|}^{2}+\frac{\upsilon_{2}}{2}{\left\|q_{l}^{\prime }-q_{l}+\omega_{2}\right\|}^{2}, \end{array}$$

where $\omega_{1}$ and $\omega_{2}$ are the error variables corresponding to (27) and (28), respectively, which function as Lagrange multipliers during the ADMM iterative process; $\upsilon_{1}$ and $\upsilon_{2}$ are the penalty factors. Regarding the primal variables $\left\{q_{l},q_{l}^{*},q_{l}^{\prime }\right\}$, they can be partitioned into two distinct groups, namely $q_{l}$ and the set $\left\{q_{l}^{*},q_{l}^{\prime }\right\}$. Based on this partitioning, the augmented Lagrangian function is also separable. Consequently, (29) can be solved by iteratively updating these two groups of optimization variables. The associated ADMM algorithm procedure primarily consists of the following three steps:

- *Update*$\left\{q_{l}^{*},q_{l}^{\prime }\right\}$: Based on the aforementioned definitions of the variables $\left\{q_{l}^{*},q_{l}^{\prime }\right\}$, their optimization can be processed separately and independently. The optimization procedure for each parameter can be formulated as
  (31) $$\begin{array}{cc} & \min_{\left\{q_{l}^{*}\right\}}\frac{\upsilon_{1}}{2}{\left\|q_{l}^{*}-q_{l}^{n}+\omega_{1}^{n}\right\|}^{2}\\ & s.t.t_{l,i}^{a}+T_{l}^{*}+t_{l(i,j)}^{*f}\leq t_{l,j}^{a},i,j\in N_{l},l\in L, \end{array}$$
  (32) $$\begin{array}{cc} & \min_{\left\{q_{l}\right\}}\frac{\upsilon_{2}}{2}{\left\|q_{l}^{\prime }-q_{l}^{n}+\omega_{2}^{n}\right\|}^{2}\\ & s.t.\sum_{i=0}^{n_{l}}\sum_{j=0}^{n_{l}}\rho_{l(i,j)}\left(T_{l}^{\prime }+t_{l,(i,j)}^{\prime f}\right)\leq t_{l}^{\max}. \end{array}$$
  where $q_{l}^{n}$, $\omega_{1}^{n}$, $\omega_{2}^{n}$ represent the solutions obtained after the n-th iteration. Due to the influence of constraints, (31) constitutes a non-convex problem. To resolve this issue, we introduce auxiliary variables $\delta_{1}=\left\{\delta_{l}^{DL},l\in L\right\}$, $\delta_{2}=\left\{\delta_{i},i\in n_{l}+1\right\}$, and $\delta_{3}=\left\{\delta_{l}^{UL},l\in L\right\}$, corresponding to the transmission rates $r_{l}^{DL}$, $r_{i}$, and $r_{l}^{UL}$, respectively. Thus, (31) can be reformulated as
  (33) $$\begin{array}{cc} \min_{\left\{q_{l}^{*},\delta_{1},\delta_{2},\delta_{3}\right\}} & \frac{\upsilon_{1}}{2}{∥q_{l}^{*}-q_{l}^{n}+\omega_{1}^{n}∥}^{2}\\ s.t. & t_{l,i}^{a}+T_{l}^{*}+t_{l(i,j)}^{*f}\leq t_{l,j}^{a},i,j\in N_{l},l\in L,\\ & \delta_{l}^{DL}\leq r_{l}^{DL^{*}},l\in L,\\ & \delta_{i}\leq r_{i}^{*},i\in N_{l},\\ & \delta_{l}^{UL}\leq r_{l}^{UL^{*}},l\in L, \end{array}$$
  where $r_{l}^{DL^{*}}$, $r_{i}^{*}$, and $r_{l}^{UL^{*}}$ represent the transmission rates after substituting $q_{l}$. However, since $r_{l}^{DL^{*}}$, $r_{i}^{*}$, and $r_{l}^{UL^{*}}$ remain non-convex with respect to $q_{l}^{*}$, (33) is still intractable for direct solution. Notably, $r_{l}^{DL^{*}}$ and $r_{l}^{UL^{*}}$ exhibit convexity with respect to $\|q_{l}^{*}-q_{0}{\|}^{2}$, while $r_{i}^{*}$ is convex with respect to $\|q_{l}^{*}-q_{i}{\|}^{2}$. To address this, we seek convex lower bounds for $R_{l}^{DL^{*}}$, $R_{i}^{*}$, and $R_{l}^{UL^{*}}$ to replace the original non-convex functions, thereby transforming the optimization problem into a convex problem. Assuming the current iteration point is $q_{l}^{*n}$, the convex lower bounds for $r_{l}^{DL^{*}}$, $r_{i}^{*}$, and $r_{l}^{UL^{*}}$ obtained via first-order Taylor expansion at $q_{l}^{*n}$ can be expressed as
  (34) $$\begin{array}{cc} \; & r_{l}^{DL^{*}}\geq B_{l}\log_{2}\left(1+\frac{ph_{l,0}}{\sigma^{2}\left(\right\|q_{l}^{*n}-q_{0}{\|}^{2}+H^{2})}\right)\\ \; & -\frac{B_{l}ph_{l,0}\left(\right\|q_{l}^{*}-q_{0}{\|}^{2}-\|q_{l}^{*n}-q_{0}{\|}^{2})}{\ln\left(2\right)(\|q_{l}^{*n}-q_{0}{\|}^{2}+H^{2})\left(\sigma^{2}\left(\right\|q_{l}^{*n}-q_{0}{\|}^{2}+H^{2})+ph_{l,0}\right)}, \end{array}$$
  (35) $$\begin{array}{cc} \; & r_{i}^{*}\geq B\log_{2}\left(1+\frac{p_{l}h_{l,i}}{\sigma^{2}\left(\right\|q_{l}^{*n}-q_{i}{\|}^{2}+H^{2})}\right)\\ \; & -\frac{Bp_{l}h_{l,i}\left(\right\|q_{l}^{*}-q_{i}{\|}^{2}-\|q_{l}^{*n}-q_{i}{\|}^{2})}{\ln\left(2\right)(\|q_{l}^{*n}-q_{i}{\|}^{2}+H^{2})\left(\sigma^{2}\left(\right\|q_{l}^{*n}-q_{i}{\|}^{2}+H^{2})+ph_{l,i}\right)}, \end{array}$$
  (36) $$\begin{array}{cc} \; & r_{l}^{UL^{*}}\geq B_{l}\log_{2}\left(1+\frac{p_{l}h_{l,0}}{\sigma^{2}\left(\right\|q_{l}^{*n}-q_{0}{\|}^{2}+H^{2})}\right)\\ \; & -\frac{B_{l}p_{l}h_{l,0}\left(\right\|q_{l}^{*}-q_{0}{\|}^{2}-\|q_{l}^{*n}-q_{0}{\|}^{2})}{\ln\left(2\right)(\|q_{l}^{*n}-q_{0}{\|}^{2}+H^{2})\left(\sigma^{2}\left(\right\|q_{l}^{*n}-q_{0}{\|}^{2}+H^{2})+ph_{l,0}\right)}, \end{array}$$
  Substituting (34)–(36) into (33) transforms the latter into a convex optimization problem, which can be efficiently solved using conventional convex optimization methods. Similarly, (32) can be solved following an analogous procedure, the details of which are omitted here for brevity.
- *Update* $q_{l}$: The optimization procedure for $q_{l}$ can be formulated as
  (37) $$\begin{array}{cc} \; & \min_{q_{l}}\sum_{l=1}^{L}\sum_{i=0}^{n_{l}}\sum_{j=0}^{n_{l}}\rho_{l(i,j)}\left(T_{l}+t_{l(i,j)}^{f}\right)\\ \; & +\frac{\upsilon_{1}}{2}\|q_{l}^{*n+1}-q_{l}+\omega_{1}^{n}{\|}^{2}+\frac{\upsilon_{2}}{2}{\left\|q_{l}^{\prime n+1}-q_{l}+\omega_{2}^{n}\right\|}^{2}, \end{array}$$
  Following the same solution approach applied to (31), we introduce auxiliary variables $\zeta_{1}=\left\{\zeta_{l}^{DL},l\in L\right\}$, $\zeta_{2}=\left\{\zeta_{i},i\in N_{l}\right\}$, and $\zeta_{3}=\left\{\zeta_{l}^{UL},l\in L\right\}$ corresponding to the transmission rates $r_{l}^{DL}$, $r_{i}$, and $r_{l}^{UL}$, respectively. Thus, (37) can be reformulated as
  (38) $$\begin{array}{cc} \min_{q_{l},\zeta_{1},\zeta_{2},\zeta_{3}} & \sum_{l=1}^{L}\sum_{i=0}^{n_{l}}\sum_{j=0}^{n_{l}}\rho_{l(i,j)}\left(T_{l}+t_{l(i,j)}^{f}\right)\\ \; & +\frac{\upsilon_{1}}{2}\|q_{l}^{*n+1}-q_{l}+\omega_{1}^{n}{\|}^{2}+\frac{\upsilon_{2}}{2}{\left\|q_{l}^{\prime n+1}-q_{l}+\omega_{2}^{n}\right\|}^{2},\\ s.t. & \zeta_{l}^{DL}\leq r_{l}^{DL^{\prime }},l\in L,\\ \; & \zeta_{i}\leq r_{i}^{\prime },i\in N_{l},\\ \; & \zeta_{l}^{UL}\leq r_{l}^{UL^{\prime }},l\in L. \end{array}$$
  The analytical approach for $r_{l}^{DL^{\prime }}$, $r_{i}^{\prime }$, and $r_{l}^{UL^{\prime }}$ follows the same methodology applied to $r_{l}^{DL^{*}}$, $r_{i}^{*}$, and $r_{l}^{UL^{*}}$ and thus will not be reiterated here. After this reformulation, (38) becomes a convex optimization problem that can be efficiently solved using standard convex optimization techniques.
- *Updating the Lagrange multiplier:* The optimization procedure for the Lagrange multiplier can be formulated as
  (39) $$\omega_{1}^{n+1}=\omega_{1}^{n}+q_{l}^{*n+1}-q_{l}^{n+1},$$
  (40) $$\omega_{2}^{n+1}=\omega_{2}^{n}+q_{l}^{\prime n+1}-q_{l}^{n+1}.$$
  The detailed procedure of addressing the problem of optimizing the UAV arrival times and hovering coordinates given fixed client clustering and task allocation strategies is presented in Algorithm 2.

**Algorithm** **2** ADMM-based UAV optimization algorithm for a given clustering strategy.**Input:** The joint strategy of the *L* clusters: $s=\{s_{1},s_{2},\ldots ,s_{L}\}$; *B*, $B_{l}$;
**Output:** Optimal UAV arrival time *t* and hovering coordinates $q_{l}$;
1:Initialize the Lagrange multiplier $\omega_{1}=0$, $\omega_{2}=0$;2:Initialize the iteration index I=0;3:Initialize the inner-iteration index n=0;4:**Repeat iteration**5:**for** each cluster l∈L **do**6:   Optimize the UAV arrival time *t* by solving (24);7:   Introduce two auxiliary variables $q_{l}^{*}$, $q_{l}^{\prime }$ and reformulate (15), (16) as (27),(28);8:   Reformulate (25) into the equivalent ADMM form;9:   **while** $n<n_{set}$ **do**10:     n=n+111:     Obtain $\left\{q_{l}^{*n},q_{l}^{\prime n}\right\}$ by solving (31)–(33);12:     Obtain $\left\{q_{l}\right\}$ by solving (37);13:     Update the Lagrange multiplier $\omega_{1}^{n}$, $\omega_{2}^{n}$ by calling (39), (40);14:   **end while**15:**end for**16:$q_{l}^{I}\leftarrow q_{l}^{n_{set}}$;17:I=I+1;18:**until** $I=I_{set}$19:**return** Optimal UAV arrival time *t* and hovering coordinates $q_{l}$


### 3.4. Overview of Clustering-Based Distributed Task Offloading Algorithms

Based on the optimization objective in (17), the utility of cluster *l* can be defined as follows:

(41) $$\phi_{l}\left(s_{l},s_{-l}\right)=\alpha T_{l}+\left(1-\alpha \right)t_{l}^{f},$$

where $t_{l}^{f}=\sum_{i=0}^{n_{l}}\sum_{j=0}^{n_{l}}\rho_{l(i,j)}t_{l(i,j)}^{f}$ denotes the flight time of UAV *l* under the current offloading link. α denotes the penalty factor. Considering the other clusters’ policy profiles $s_{-l}$, each cluster updates its policy by selecting policy $s_{l}^{\prime }$ in order to

(42) $$s_{l}^{\prime }={\mathrm{argmax}}_{s_{l}^{\prime }\in S_{l}}A_{l}\left(s_{l}^{\prime },s_{-l}\right).$$

The algorithm begins by initializing a network consisting of *L* TCs, *C* ACs, *L* UAVs, and a TBS. Initially, each cluster selects a clustering strategy $s_{l}$ through BFS-based game clustering before evaluating its utility $A_{l}\left(s_{l},s_{-l}\right)$. Next, each cluster chooses a best response strategy [27] to maximize its utility. If the newly chosen strategy provides higher utility than the existing one, it will replace the existing strategy. These steps are repeated until no cluster can improve its utility by updating its strategy. The detailed procedure is presented in Algorithm 3.
**Algorithm** **3** Overview of clustering-based distributed task offloading algorithms.**Input:** Clustering strategy: $s_{l}$; **Output:** Optimal global latency *T*;
1:Initialize the last selected split layer m=0;2:Initialize the iteration index k=0;3:Initialize the decision-making process for each TC by assigning each user a randomly selected strategy from the available strategy space;4:**Repeat iteration**5:**for** each cluster l∈L **do**6:   Calculate the global utility as negative reward by calling Algorithms 1 and 2;7:   **if** $A_{l}\left(s_{l},s_{-l}\right)<A_{l}\left(s_{l}^{\prime },s_{-l}\right)$ **then**8:     **Let** $s_{l}\left(k+1\right)=s_{l}$;9:   **else**10:     **Let** $s_{l}\left(k+1\right)=s_{l}\left(k\right)$11:   **end if**12:**end for**

### 3.5. Convergence and Complexity Analysis

In this subsection, we conduct a brief convergence and computational complexity analysis of the proposed ADTO framework during the training process. The solution obtained from NE is not necessarily optimal. We use the Price of Anarchy (PoA) to quantify the worst-case scenario of NE. As described in Definition 2, let $s^{*}=\left\{s_{1}^{*},\ldots ,s_{L}^{*}\right\}$ be an NE strategy profile and $A_{l}\left(s_{l}^{*},s_{-l}^{*}\right)$ denote maximizing the utility; then, the PoA can be expressed as

(43) $$PoA=\frac{max_{s_{l}^{*}\in s^{*}}\sum_{l\in L}A_{l}\left(s_{l}^{*},s_{-l}^{*}\right)}{min_{s_{l}\in S_{l}}\sum_{l\in L}A_{l}\left(s_{l},s_{-l}\right)}.$$

**Theorem** **2.** *The maximum value of the PoA for the distributed USV clustering game is*

(44) $$\frac{\sum_{l\in L}\left(t_{l}^{DL}+t_{l}^{local}+t_{l}^{UL}+t_{l}^{f}\right)}{\sum_{l\in L}min\left(T_{l}^{local},T_{l}^{D2D}\right)}.$$

**Proof.**  Under the NE, the overall delay for each TC *l* to complete the global task $Q_{l}$ is at most $t_{l}^{DL}+t_{l}^{local}+t_{l}^{UL}+t_{l}^{f}$, corresponding to the strategy in which the TC always executes its computation tasks locally. Hence, we obtain

(45) $$\begin{array}{cc} \sum_{l\in L}A_{l}\left(s_{l}^{*},s_{-l}^{*}\right) & \leq A_{l}^{local}\left(s_{l},s_{-l}\right)\\ & =-\sum_{l\in L}\left(t_{l}^{DL}+t_{l}^{local}+t_{l}^{UL}+t_{l}^{f}\right). \end{array}$$

(46) $$\begin{array}{cc} \;\;\;\;\;\;\;\;\;\;\;\;\;\;\;\;\;\;\sum_{l\in L}A_{l}\left(s_{l},s_{-l}\right) & \geq min\left(A_{l}^{local},A_{l}^{D2D}\right)\\ & =min\left(-T_{l}^{local}+t_{l,local}^{f},-T_{l}^{D2D}+t_{l,D2D}^{f}\right). \end{array}$$

According to (45) and (46), we can derive the upper bound of the PoA:

(47) $$PoA\leq \frac{\sum_{l\in L}\left(t_{l}^{DL}+t_{l}^{local}+t_{l}^{UL}+t_{l}^{f}\right)}{\sum_{l\in L}min\left(T_{l}^{local}+t_{l,local}^{f},T_{l}^{D2D}+t_{l,D2D}^{f}\right)}.$$

□

Theorem 2 establishes that even if the algorithm converges to the worst NE, the overall optimization objective does not deteriorate arbitrarily. Crucially, even in the worst-case scenario, its performance remains consistently within a constant factor of the centralized optimal solution.

The computational complexity of BFS-based distributed game clustering primarily depends on the number of edges *E* and nodes *V* in the graph. Thus, the computational complexity of BFS-based distributed game clustering can be expressed as O(|V|+|E|). The computational complexity of Algorithm 1 is denoted as $O(n_{l})$, while the computational complexity of Algorithm 2 is denoted as $O(I\cdot L\cdot n_{set}\cdot n_{l})$, where *I* represents the number of iterations in Algorithm 2. After omitting constant terms, the overall complexity of ADTO is derived as $T(|V|+|E|+n_{l}+I\cdot L\cdot n_{set}\cdot n_{l})$. After omitting the constant term, the overall computational complexity can be expressed as $T(|V|+|E|+L\cdot n_{l})$. In summary, the computational complexity of ADTO is acceptable, and this design is well suited to scenarios with high real-time performance requirements and limited resources.

## 4. Experimental Results and Analysis

In this section, we implement simulations of the proposed framework using Python 3.9 and PyTorch 11.3. Meaningful results from extensive experiments are presented to validate the effectiveness of the developed solution.

### 4.1. Parameter Settings

We consider a sector-shaped area centered at the TBS, which defines the free flight area of the UAVs. This area contains multiple ACs and TCs, totaling 12 nodes. Since each UAV acts as a CH, the number of UAVs equals the number of TCs. The sector radius is 500 m, and thus, the coordinates of the TBS and the UAV charging station are $q_{0}=\left(0,0\right)$. For D2D communication links between clients *i* and *j*, we configure all clients with an identical transmission power of 24 dBm. The noise variance $\sigma^{2}=-110$ dBm and the channel power gain is −60 dB, which are constant in all phases. The flying altitude *H* of the UAV is set to 100 m, and the flight speed is set to 20 m/s. For each TC, the global task data size is 15 Mbits. For all USVs, the required CPU cycles per bit are uniformly distributed in [1500, 2000] cycles/bit. The computing resources for the TC and AC are configured as $f_{t}\in \left[0.5,2\right]\times 10^{9}$ CPU cycles/s and $f_{a}\in \left[6,10\right]\times 10^{9}$ CPU cycles/s, respectively [23]. For the TBS, its transmit power *P* is 46 dBm, the default communication bandwidth is 5 MHz, and the computing capability is $f_{tbs}=40\times 10^{9}$ CPU cycles/s. The penalty factor α=0.9.

### 4.2. Convergence Behavior

**Figure 3:**
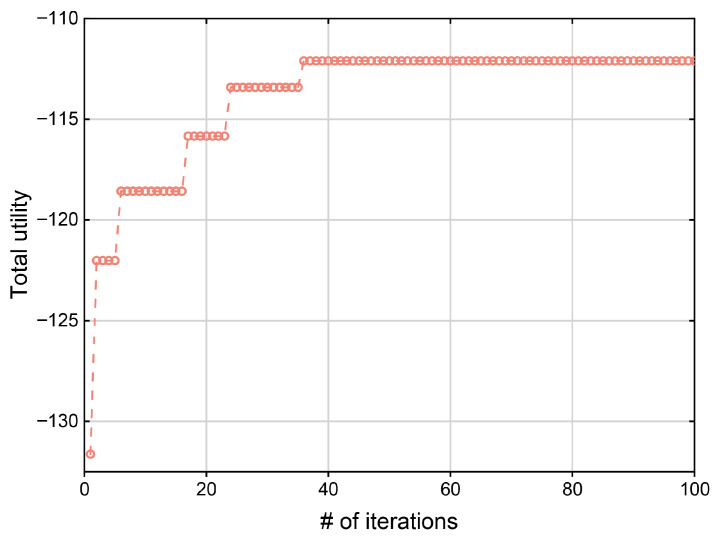
The convergence of the total utility.

We first analyze the convergence properties of the ADTO algorithm under a configuration of 6 TCs and 6 ACs. As illustrated in Figure 3, 500 independent trials were conducted under identical topological conditions. Consistent with theoretical expectations, the global utility increased with iteration count and converged after approximately 38 iterations. When combined with the theoretical analyses of Theorems 1 and 2, these experimental results confirm that ADTO exhibits robust convergence behavior and attains stable solutions within finite iterations. The penalty factor α is set to 0.9.

### 4.3. Task Execution Latency Comparison

#### 4.3.1. Local Offloading Mechanisms

We selected several advanced local offloading algorithms for comparison with ADTO, and the details of the selected algorithms are described below.

**Random offloading:** The random offloading approach randomly assigns the size of subtasks to the USVs within the cluster. This methodology fails to ensure optimal task assignment to the most suitable nodes, consequently inducing suboptimal system performance.

**Greedy:** The greedy approach offloads the entire task to the nearest and most computationally powerful AC without task splitting.

**MAC-L:** This approach models complex computation tasks as “task flows” with dependencies and employs the multi-actors-critic (MAC)-based MARL approach. For the purpose of comparison, only the local latency is considered here.

**Figure 4:**
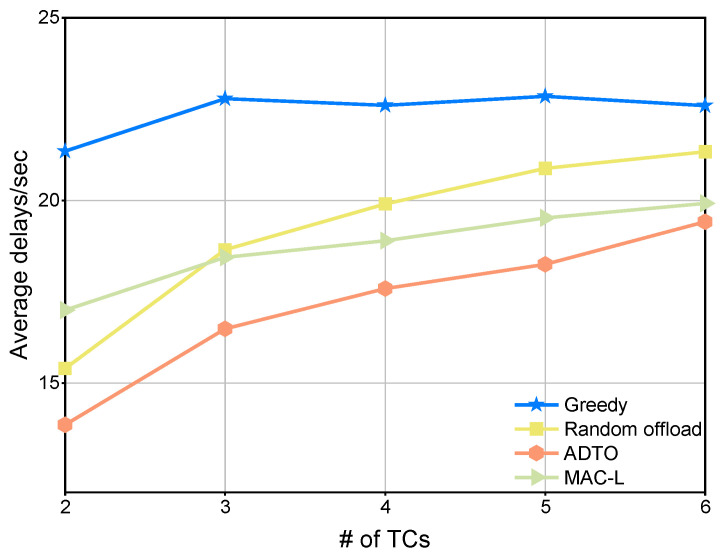
Comparison of local performance under four mechanisms for varying number of TCs.

Figure 4 compares the ADTO algorithm against three intra-cluster offloading schemes. As the number of TCs increases, the greedy method is only marginally affected, whereas the other three methods exhibit a clear upward trend in the delay. Nevertheless, despite its relative insensitivity to TC count, the greedy approach consistently incurs the highest latency among the four. In contrast, ADTO delivers the lowest delay across all tested TC levels.

**Figure 5:**
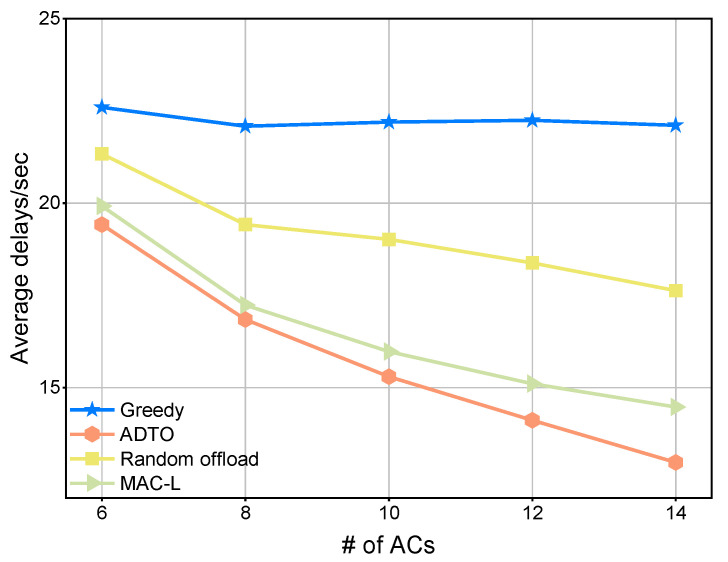
Comparison of local performance under four mechanisms for varying number of ACs.

Figure 5 depicts the performance of the three schemes as the number of ACs grows, with the number of TCs held constant at 6. Because the greedy algorithm simply offloads the entire task to the nearest AC with the greatest computational capacity, increasing the number of ACs does not significantly improve its overall performance. The random offload approach is further hampered by resource conflicts and thus underperforms relative to ADTO. Furthermore, MAC-L is the method among the three that has the closest performance to that of ADTO. This is because as the number of ACs increases, the impact of resource conflicts on the task scheduling of MAC-L gradually diminishes. Therefore, ADTO remains the most performant method among the four.

#### 4.3.2. Global Offloading Mechanisms

Next, we selected several advanced local offloading algorithms for comparison with ADTO, and the details of the selected algorithms are described below.

**Global local computing (GLC):** After receiving the TBS task transmitted via the UAV, each TC selects to execute the task locally.

**Global edge offloading (GEO):** Based on Algorithm 1, when the local TC receives the TBS task transmitted via the UAV, it decides to offload part of the task to the TBS for execution.

**MAC-L:** The MAC-L method takes into account the communication delay while remaining the same as the previous method.

**Figure 6:**
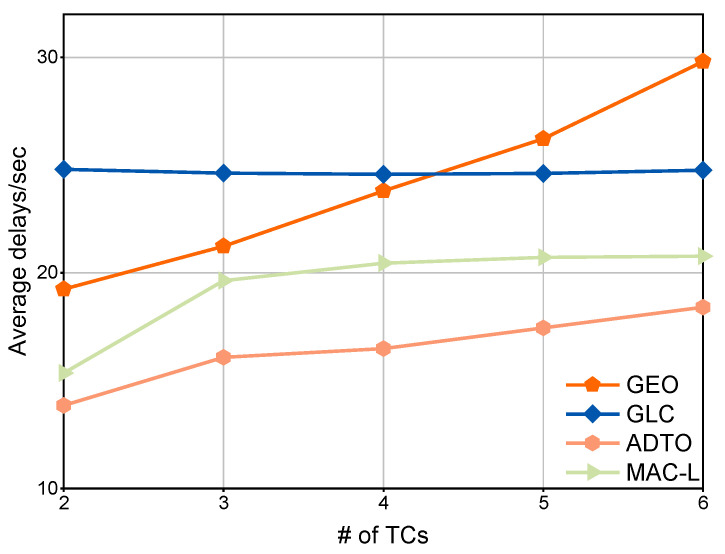
Comparison of global performance under four mechanisms for varying number of TCs.

We conducted a comparative evaluation of the ADTO algorithm against three other global offloading mechanisms. As shown in Figure 6, the average latency of all four schemes increases as the number of TCs grows. However, the GEO algorithm’s latency rises sharply due to its sensitivity to constrained communication resources. In contrast, the GLC scheme exhibits relatively minor fluctuations but registers the highest average delay when fewer than four TCs are present. As a scheduling algorithm, MAC-L exhibits increasing latency when the number of TCs rises and the number of ACs relatively decreases. This degradation stems from exacerbated scheduling complexity when allocating TC tasks across diminished computational resources. In contrast, ADTO consistently outperforms all three baseline methods, maintaining the lowest observed latency across all evaluated conditions.

**Figure 7:**
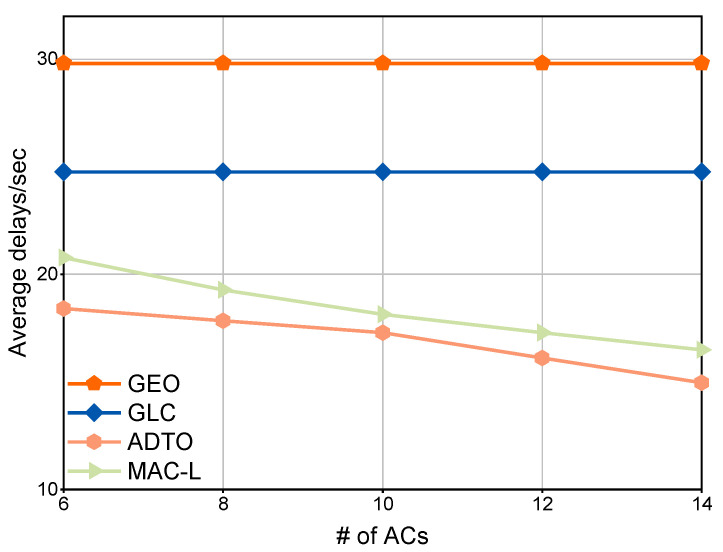
Comparison of global performance under four mechanisms for varying number of ACs.

Figure 7 illustrates a global performance comparison of the three mechanisms as the number of ACs varies. Because the performance of both GEO and GLC is independent of AC count, their curves appear as horizontal lines. In contrast, the total latency under ADTO decreases with an increasing number of ACs. This declining trend does not continue indefinitely as AC numbers grow since a balance point will be reached where the intra-cluster communication and computing overhead offsets the benefit gained from adding more ACs.

### 4.4. UAV Time Cost Comparison

Finally, we compare the latency performance of four schemes: (1) our proposed D2D offloading method, integrating Algorithms 1 and 2, denoted by ADTO; (2) the ADTO method which uses UAV-assisted computation offloading and where the UAV undertakes the computation task, denoted by ADTO-U; (3) the ADTO method with the UAV hover coordinates fixed at the horizontal coordinates of the served USV, denoted by ADTO-F; and (4) the ADTO method with tasks partially and randomly offloaded to neighboring USVs, denoted by ADTO-R.

**Figure 8:**
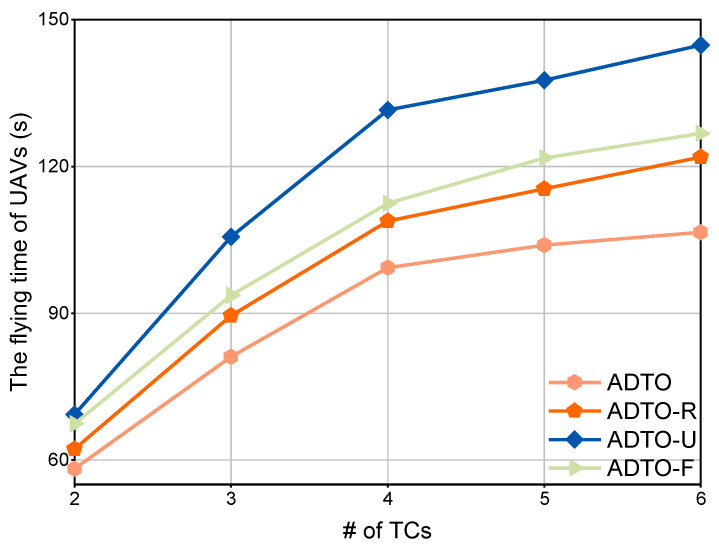
Comparison of the UAV flying time for varying numbers of ACs.

Figure 8 presents the variations in UAV flight time for different schemes as the number of TCs increases. It can be observed that across all schemes, UAV flight time correspondingly rises with an increasing number of TCs. Under the same number of TCs, ADTO consistently achieves the shortest flight time. Specifically, for ADTO-F, its hovering coordinates are fixed, which results in a longer flight distance compared to that of ADTO. Regarding ADTO-U, the UAV is required to perform local computational tasks, necessitating closer proximity to the TCs. Additionally, it is constrained by battery capacity, leading to frequent travel between the TBS and TCs. Finally, although ADTO-R is the closest scheme to ADTO and has a relatively shorter flight time compared to that of the other two schemes, the introduction of randomness prevents it from guaranteeing an optimal UAV flight trajectory.

## 5. Conclusions

A novel distributed computation offloading framework for maritime IoT systems has been proposed in this paper. Considering practical communication constraints and real-world application demands in shipping operations, the proposed framework employs potential game theory to model the client clustering optimization problem as a distributed computation offloading game. This approach aims to maximize the overall offloading efficiency while simultaneously addressing communication limitations and enhancing resource utilization. Building upon this, we formulate a global latency minimization problem that jointly optimizes UAV hovering coordinates and arrival times. This problem is solved by decomposing it into subproblems addressed via a joint ADMM and SCA approach, effectively reducing the time between UAV arrivals and hovering coordinates. Extensive simulation results validate that the framework can effectively reduce the overall system delay while making full use of the available computing resources.

Although the distributed computational offloading framework proposed in this paper demonstrates significant effectiveness and outperforms several prevalent schemes in reducing global latency, there remain opportunities for further improvements. Firstly, as the number of TCs increases, the performance of ADTO exhibits a declining trend. Introducing Digital Twin (DT) technology can provide a novel approach to ensuring quality of service in regions with high-density data centers and efficiently offloading complex tasks [28]. Secondly, considering the movement characteristics of USVs, future research directions could include advanced positioning techniques such as particle filter-based Received Signal Strength (RSS) localization, multi-agent Q-learning methods for trajectory tracking [29], and multi-agent reinforcement learning (MARL) methods for dynamic resource allocation [30]. Furthermore, the trustworthiness of D2D computational offloading has become a critical research area. While offloading tasks from TCs to adjacent ACs alleviates computational deficits of individual TCs, it simultaneously introduces risks of erroneous computations or malicious interference from untrusted nodes. The Fuzzy Comprehensive Trust Evaluation (FCTE) algorithm, which has proven effective in vehicular edge computing scenarios, represents a viable solution for enhancing offloading security and could serve as a potential approach to improving overall offloading safety [31].

## Data Availability

The data is not publicly available due to confidentiality agreements with the data provider.

## Abbreviations

The following abbreviations are used in this manuscript:

UAV	unmanned aerial vehicle
USV	unmanned surface vessel
D2D	device-to-device
BFS	breadth-first search
ADMM	Alternating Direction Method of Multipliers
MEC	mobile edge computing
SCA	Successive Convex Approximation
IoT	Internet of Things
CH	cluster head
CSI	Channel State Information
LoS	Line of Sight
TC	task client
AC	assisting client
EPG	exact potential game
NE	Nash Equilibrium
RSS	Received Signal Strength
MARL	multi-agent reinforcement learning
FCTE	Fuzzy Comprehensive Trust Evaluation
DT	Digital Twin
TBS	terrestrial base station
